# The role of apoptosis repressor with a CARD domain (ARC) in the therapeutic resistance of renal cell carcinoma (RCC): the crucial role of ARC in the inhibition of extrinsic and intrinsic apoptotic signalling

**DOI:** 10.1186/s12964-017-0170-5

**Published:** 2017-05-02

**Authors:** Csaba Toth, Sarah Funke, Vanessa Nitsche, Anna Liverts, Viktoriya Zlachevska, Marcia Gasis, Constanze Wiek, Helmut Hanenberg, Csaba Mahotka, Peter Schirmacher, Sebastian Heikaus

**Affiliations:** 1 Institute of Pathology, Heinrich Heine University Hospital, Medical Faculty, Moorenstrasse 5, 40225 Düsseldorf, Germany; 2 Institute of Pathology, University Hospital Heidelberg, Im Neuenheimer Feld 224, 69120 Heidelberg, Germany; 3 Department of Neurology, Heinrich Heine University Hospital, Medical Faculty, Moorenstrasse 5, 40225 Düsseldorf, Germany; 4 Department of Otorhinolaryngology, Head and Neck Surgery, Heinrich Heine University, Universitätsstrasse 1, 40225 Düsseldorf, Germany; 5 Department of Pediatrics, the Herman B. Wells Center for Pediatric Research 702 Barnhill Dr, Indianapolis, IN 46202 USA

**Keywords:** ARC, Apoptosis, Bcl-2 family, renal cell carcinoma (RCC), ABT-263, TRAIL

## Abstract

**Background:**

Renal cell carcinomas (RCCs) display broad resistance against conventional radio- and chemotherapies, which is due at least in part to impairments in both extrinsic and intrinsic apoptotic pathways. One important anti-apoptotic factor that is strongly overexpressed in RCCs and known to inhibit both apoptotic pathways is ARC (apoptosis repressor with a CARD domain).

**Methods:**

Expression and subcellular distribution of ARC in RCC tissue samples and RCC cell lines were determined by immunohistochemistry and fluorescent immunohistochemistry, respectively. Extrinsic and intrinsic apoptosis signalling were induced by TRAIL (TNF-related apoptosis-inducing ligand), ABT-263 or topotecan. ARC knock-down was performed in clearCa-12 cells using lentiviral transduction of pGIPZ. shRNAmir constructs. Extrinsic respectively intrinsic apoptosis were induced by TRAIL (TNF-related apoptosis-inducing ligand), ABT263 or topotecan. Potential synergistic effects were tested by pre-treatment with topotecan and subsequent treatment with ABT263. Activation of different caspases and mitochondrial depolarisation (JC-1 staining) were analysed by flow cytometry. Protein expression of Bcl-2 family members and ARC in RCC cell lines was measured by Western blotting. Statistical analysis was performed by Student’s *t*-test.

**Results:**

Regarding the extrinsic pathway, ARC knockdown strongly enhanced TRAIL-induced apoptosis by increasing the activation level of caspase-8. Regarding the intrinsic pathway, ARC, which was only weakly expressed in the nuclei of RCCs in vivo, exerted its anti-apoptotic effect by impairing mitochondrial activation rather than inhibiting p53. Topotecan- and ABT-263-induced apoptosis was strongly enhanced following ARC knockdown in RCC cell lines. In addition, topotecan pre-treatment enhanced ABT-263-induced apoptosis and this effect was amplified in ARC-knockdown cells.

**Conclusion:**

Taken together, our results are the first to demonstrate the importance of ARC protein in the inhibition of both the extrinsic and intrinsic pathways of apoptosis in RCCs. In this context, ARC cooperates with anti-apoptotic Bcl-2 family members to exert its strong anti-apoptotic effects and is therefore an important factor not only in the therapeutic resistance but also in future therapy strategies (i.e., Bcl-2 inhibitors) in RCC. In sum, targeting of ARC may enhance the therapeutic response in combination therapy protocols.

## Background

Renal cell cancer (RCC) shows strong resistance to conventional chemotherapy, especially those with Bcl-2 overexpression which have even worse prognosis and poorer therapeutic response. Downregulation of Bcl-2 increased chemosensitivity in clinical studies in a wide variety of cancers. In RCC cells the Bcl-2 inhibition combined with cisplatin exerts the therapeutic effects of cisplatin providing an attractive therapeutic strategy in Bcl-2 overexpressing RCCs. Despite therapeutic efforts, RCC remains highly resistant to systemic chemotherapy [1].

Apoptosis repressor with a caspase recruitment domain (ARC) is a potent inhibitor of apoptosis that it is strongly expressed in multiple terminally differentiated cells (i.e., ganglion cells, skeletal muscle and heart muscle) [2, 3] as well as solid cancers such as carcinomas, melanomas, and gliomas [4–10]. Different expression levels of ARC have been already observed in different cell lines (MCF-7 - breast cancer, A-549 - non-small lung cancer, HT-29 - colon cancer, PC-3 prostate cancer, A-498 - kidney cancer). ARC level was different not only in different cancer cell types, but also among cell types of same cancer types [11]. While ARC confers significant beneficial effects in terminally differentiated cells, such as the attenuation of myocardial ischemia in cardiomyocytes [12], neuroprotection [13] and the prevention of acute liver failure [14, 15], its anti-apoptotic properties in malignant tumours are detrimental because they protect against activation of extrinsic as well as intrinsic apoptotic signals. ARC is a unique protein inhibiting both the extrinsic (death receptor mediated) and intrinsic (mitochondrial/ER stress induced) apoptotic pathways. ARC can inhibit apoptosis almost independently from the inducing cause, such as death receptor activation, hypoxia, hydrogen peroxide, oxidative stress, serum deprivation, ischaemic reperfusion, doxorubicin or γ-radiation [3, 8, 11, 16, 17]. The fact that ARC inhibits both, extrinsic and intrinsic apoptotic pathways interacting with them in a non-homotypic death-fold manner [16], can provide a growth advantage to cancer cells. In addition, high level of ARC protein in breast cancer cells is associated with chemo- and radioresistance [8, 11].

ARC with its CARD binds to death receptors, Fas, FADD and pro-caspase-8 and inhibits the assembly of DISC, thus abrogating the extrinsic apoptotic signaling. In the extrinsic pathway of apoptosis, ARC can directly bind and inhibit caspase-8 [3], whereas in the intrinsic pathway, ARC interacts with nuclear p53 to prevent p53 tetramerisation and induce the translocation of p53 to the cytoplasm, thereby preventing p53 activation [17].

In case of ARC knockdown, assembly of death-inducing signaling complex (DISC) will be facilitated and spontaneous Bax activation will be triggered resulted in apoptosis [8, 16]. In the cytoplasm and mitochondria, ARC also binds and inhibits caspase-2 as well as Puma, Bad and Bax, important pro-apoptotic members of the Bcl-2 family [18, 19].

Furthermore, as a result of differences in binding affinity for its interaction partners, ARC is able to modulate the activation of both the extrinsic and intrinsic pathways of apoptosis. As a result, Puma releases caspase-8 from its binding to ARC, which enables caspase-8 to induce the extrinsic apoptotic pathway [20]. In addition, the anti-apoptotic role of ARC is even more complex, as it can inhibit calcium (2+)-induced apoptosis by binding calcium and prevent the activation c-jun N-terminal kinase (JNK) [14, 21].

One novel and orally bioavailable Bcl-2 inhibitor is Navitoclax (ABT-263) acting as Bcla-3 homology 3 (BH3) mimetic. ABT-263 inhibits selectively Bcl-2, Bcl-xl and Bcl-w in a wide range of human cancer cell lines (i.e., small cell lung cancer, ALL, NHL, myeloma). Furthermore, in clinical trials, ABT-263 showed a significant anti-tumor activity as a monotherapy or in combination with conventional chemotherapeutic agents (i.e., irinotecan, erlotinib, 5-FU, paclitaxel etc.) [22, 23]. For example, ABT-263 in combination with 5-FU enhances significantly the effects of 5-FU and intensify apoptosis in oesophageal cancer cell lines. This effect can be explained by inhibition of YAP-1/SOX-9 axis and Wnt signalling [24].

Furthermore, ABT-263 and paclitaxel combination showed also a synergistic effect on both paclitaxel sensitive and resistant prostate cancer cell lines by interaction of ABT-263 and Bcl-xl in both cell lines [25]. ABT-263 disrupts Bcl-2/Bcl-xl interactions with pro-apoptotic proteins (i.e., Bim) leading to initiation of apoptosis. In addition, ABT-263 induces Bax translocation, cytochrome c release leading to apoptosis [26].

Because ARC performs important anti-apoptotic functions, we previously investigated the expression of ARC in renal cell carcinomas (RCCs) of the clear cell type, which are known to be very resistant towards chemotherapy. These findings demonstrated strong overexpression of ARC in all RCCs compared to non-neoplastic renal tissue and therefore proposed an important anti-apoptotic role for ARC in mediating the well-known resistance to apoptosis observed in RCCs [5, 27, 28]. However, it remained unclear which pathways were functionally impaired following ARC overexpression in RCCs. Specifically, the role of ARC in the inhibition of the mitochondrial pathway of apoptosis, which we and others previously found to be strongly impaired in RCCs [27, 28], became the focus of our current interest because reactivation of this pathway using recently developed targeted therapeutic strategies with Bcl-2 inhibitors may prove to be a promising therapeutic approach.

In this paper, we show for the first time that ARC is an important anti-apoptotic factor in RCCs of the clear cell renal cancer and that its effects are mediated through interference with mitochondrial apoptosis, further enhancement of the apoptosis-inhibiting properties of Bcl-2 family members and inhibition of the extrinsic pathway. Furthermore, we confirm that targeted therapy with Bcl-2 inhibitors may represent a promising new therapeutic approach for RCCs, especially in combination with DNA-damaging drugs such as topotecan or other compounds that reduce the protein expression of anti-apoptotic members of the Bcl-2 family.

## Methods

### Immunohistochemistry

Immunohistochemistry was performed using the labelled streptavidin-biotin method. A primary antibody for ARC (Table 1) was applied to the sections. Visualisation of this primary antibody was achieved following incubations with a biotinylated secondary antibody, labelled streptavidin and diaminobenzidine. Negative controls were performed by omitting the primary antibody. The tunica muscularis of the vessels was used as an internal positive control.

**Table 1:** Antibodies used for western blot analyses and immunohistochemistry with their sources, dilutions and manufacturers

Protein	Source	Dilution	Manufacturer
Bcl-A1	Mouse, monoclonal	1:500	Santa Cruz, Germany
ARC (IH)	Rabbit, poloyclonal	1:2 000	Santa Cruz, Germany
ARC (WB)	Rabbit, polyclonal	1:1 000	Thermo Scietific, Germany
β-Actin	Mouse, monoclonal	1:10 000	DAKO, Germany
Bad	Rabbit, polyclonal	1:1 000	Cell Signalling, Germany
Bak	Rabbit, polyclonal	1:1,000	Cell Signalling, Germany
Bax	Rabbit, polyclonal	1: 1 000	Cell Signalling, Germany
Bcl-2	Mouse, monoclonal	1:1 000	DAKO, Germany
Bcl-w	Rabbit, polyclonal	1:1 000	Santa Cruz, Germany
Bcl-xl	Rabbit, polyclonal	1:1 000	Cell Signalling, Germany
Bid	Rabbit, polyclonal	1:1 000	Cell Signalling, Germany
Bim	Rabbit, polyclonal	1:1 000	Cell Signalling, Germany
Bok	Rabbit, polyclonal	1:1 000	Cell Signalling, Germany
GAPDH	Rabbit, polyclonal	1:4 000	Sigma, Germany
Mcl-1	Rabbit, polyclonal	1:1 000	Cell Signalling, Germany
Phospho-p44/42 (Thr202/Tyr204)	Rabbit, polyclonal	1:1 000	Cell Signalling, Germany
PUMA	Rabbit, polyclonal	1:1 000	Cell Signalling, Germany
α-Tubulin	Mouse, monoclonal	1:10 000	Sigma, Germany
Secondary antibodies			
Rabbit	Goat, polyclonal	1:10 000	LI-COR, Germany
Mouse	Goat, polyclonal	1:10 000	LI-COR, Germany
Rabbit, AlexaFluor 514	Goat, polyclonal	1:1 000	Invitrogen, Germany
mMouse, AlexaFluor 594	Goat, polyclonal	1:1 000	Invitrogen, Germany

For semiquantitative analysis of ARC-expression cytoplasmatic staining was scored from 0 to 12 and nuclear expression was scored from 0 to 4. For scoring of the cytoplasmatic as well as nuclear ARC-expression the amount of positive cells was subdivided as follows: 0 (missing reaction in all cells), 1 (positive reaction in less than 10% of the cells), 2 (positive reaction in 10–50%), 3 (positive reaction in 50–80%) or 4 (positive reaction in more than 80%). For cytoplasmatic scoring this first numerical value was multiplied with the mean intensity of cytopasmatic ARC-staining: 1 (weak staining signal), 2 (moderate staining signal) or 3 (strong staining signal).

### Cell culture

RCC cell lines and HEK293T cells were grown at 37 °C in an atmosphere containing 5% CO_2_ in Dulbecco’s modified Eagle’s medium (DMEM) containing 10% foetal bovine serum (FCS), 2 mM glutamine, 100 U/ml penicillin and 100 μg/ml streptomycin. Transduced RCC cells were maintained in the medium described above supplemented with 2 μg/ml puromycin. For treatment, cells were exposed to the following substances dissolved in culture medium for defined periods of time: 0.1 to 10 μg/ml topotecan (Hycamtin®, GSK, Buehl, Germany), 100 ng/ml superkillerTrail (EnzoLifeScience, Lörrach, Germany) or 10 or 20 μM ABT263 (Navitoclax®, Selleckchem, Texas, USA) or 50 μM UO126 (Selleckchem). The corresponding negative controls were prepared with the appropriate solvent (PBS or DMSO).

### Lentiviral transduction and ARC knockdown with pGIPZ shRNAmir constructs

HEK 293 T cells were transfected using 45 μg polyethyleneimine (Sigma) with 5 μg of the HIV1 helper plasmid pCD/NL-BH to express HIV1 gag/pol/rev (pCD/NL-BH) [29], 5 μg of the envelope vector pczVSV-G [30] and 5 μg of the pGIPZ ARCshRNA plasmid (Thermo Scientific, Schwerte, Germany). As a control, we used the pGIPZ non-silencing shRNA plasmid carrying a scrambled shRNA. Viral supernatants were harvested 48 h after transfection, filtered and used to transduce clearCa-12 RCC cells. The selection of cells with integrated copies of the shRNA-expressing vector was accomplished using puromycin (2 μg/ml) in the culture media. TurboGFP, which is also encoded by the pGIPZ plasmid, enabled visual detection of transduced cells, and ARC knockdown was verified by western blotting (Fig. 2a).

### Assessment of cell viability

Cell viability was determined using trypan blue exclusion and the Neubauer counting chamber.

### Flow cytometry

For flow cytometry-based caspase activity assays, approximately 1x10^5^ cells were resuspended in 300 μl DMEM containing 1 μl of sulfo-rhodamine-conjugated caspase substrate (Red Caspase Staining Kit, Promokine, Heidelberg, Germany) and incubated for 0.5–1 h. After washing the cells twice, red fluorescence was detected with the flow cytometer (Partec, Muenster, Germany) in FL-2 channel. The cyanine dye JC-1-(5,5′,6,6′-tetrachloro-1,1′,3,3′-tetraethylbenzimidazolylcarbocyanine iodide) (Life Technologies, Darmstadt, Germany) was used to measure the breakdown of mitochondrial membrane potential [31]. The harvested cells were resuspended in DMEM containing 10 μg/ml JC-1 staining dye. The activation of mitochondria was observed as an increase in green fluorescence (FL-1 channel of flow cytometer). The difference between treated and control cells are reported as the percent mitochondrial activation.

### Fluorescence microscopy

RCC cells were fixed in 3.7% formaldehyde and permeabilised in PBST. Nonspecific binding was blocked by incubation with 10% normal goat serum in PBST, followed by incubation with an anti-ARC polyclonal antibody (1:500) and an anti-p53 monoclonal antibody (1:500). For detection, AlexaFluor® 594 goat anti-rabbit IgG and AlexaFluoir® 514 goat anti-mouse secondary antibodies (Life Technologies,) diluted 1:500 in blocking buffer were added to cells. During the subsequent PBST washing steps, DAPI staining was performed. Finally, slides were mounted with Vectashield mounting medium (Biozol, Echingen, Germany) and analysed with a laser scanning confocal microscope (Zeiss LSM510, Jena, Germany) equipped with an Argon/2 laser (488 nm, 514 nm), a Helium-Neon laser (549 nm) and a laser diode (405). The ZEN 2011 software (Zeiss) was used for data analysis.

### Protein extraction and western blotting

Protein extraction and western blotting were performed according to standard protocols. In short, cell lines were lysed with lysis buffer (100 mM NaCl, 10 mM Tris–HCl, pH 7.6, 1 mM EDTA, 1% NP40, protease and phosphatase inhibitors). The protein concentration of the supernatant was determined using the Bradford method (Bio-Rad, Muenchen, Germany). Protein lysate (50 μg) was separated under denaturing conditions in 10–15% polyacrylamide gels. The protein was then transferred to a nitrocellulose membrane. Detection of the proteins was performed using human-specific monoclonal or polyclonal primary antibodies prior to incubation of the membrane with the appropriate secondary antibodies (Table 1). Fluorescence was then visualised with the infrared imager ODYSSEY (LI-COR, Homburg, Germany), and densitometric analyses were performed using the Odyssey 2.1.12 Software.

### RNA extraction, reverse transcription and real-time PCR array

To analyse the influence of ARC knockdown on p53-dependent gene expression, we used the RT^2^ PCR Profiler Array (Qiagen, Hilden, Germany) according to the manufacturer’s protocol. RT was performed with the RT^2^ First Strand kit (Qiagen), and the iCycler iQ5 (Bio-Rad) was used for quantitative RT-PCR. Data analysis was performed using the ΔΔCt method and the appropriate software available on the Qiagen website. The results are presented as scatter blots. The fold-change (2^(−ΔΔCt)^) was used to quantify up- or downregulation of gene expression and was calculated for each gene represented on the array. A fold change of ±4 was defined as a statistically significant change in gene expression.

## Results

### ARC is overexpressed in the cytoplasm and nuclei of clear cell RCCs in vivo and in vitro

We previously showed that whole cell lysates from clear cell RCCs overexpress ARC at the mRNA and protein level [5]. In the present study, we sought to confirm this overexpression and to analyse possible differences in the subcellular localisation of ARC in RCCs compared to non-neoplastic renal tissue by immunohistochemistry.

**Fig. 1:**
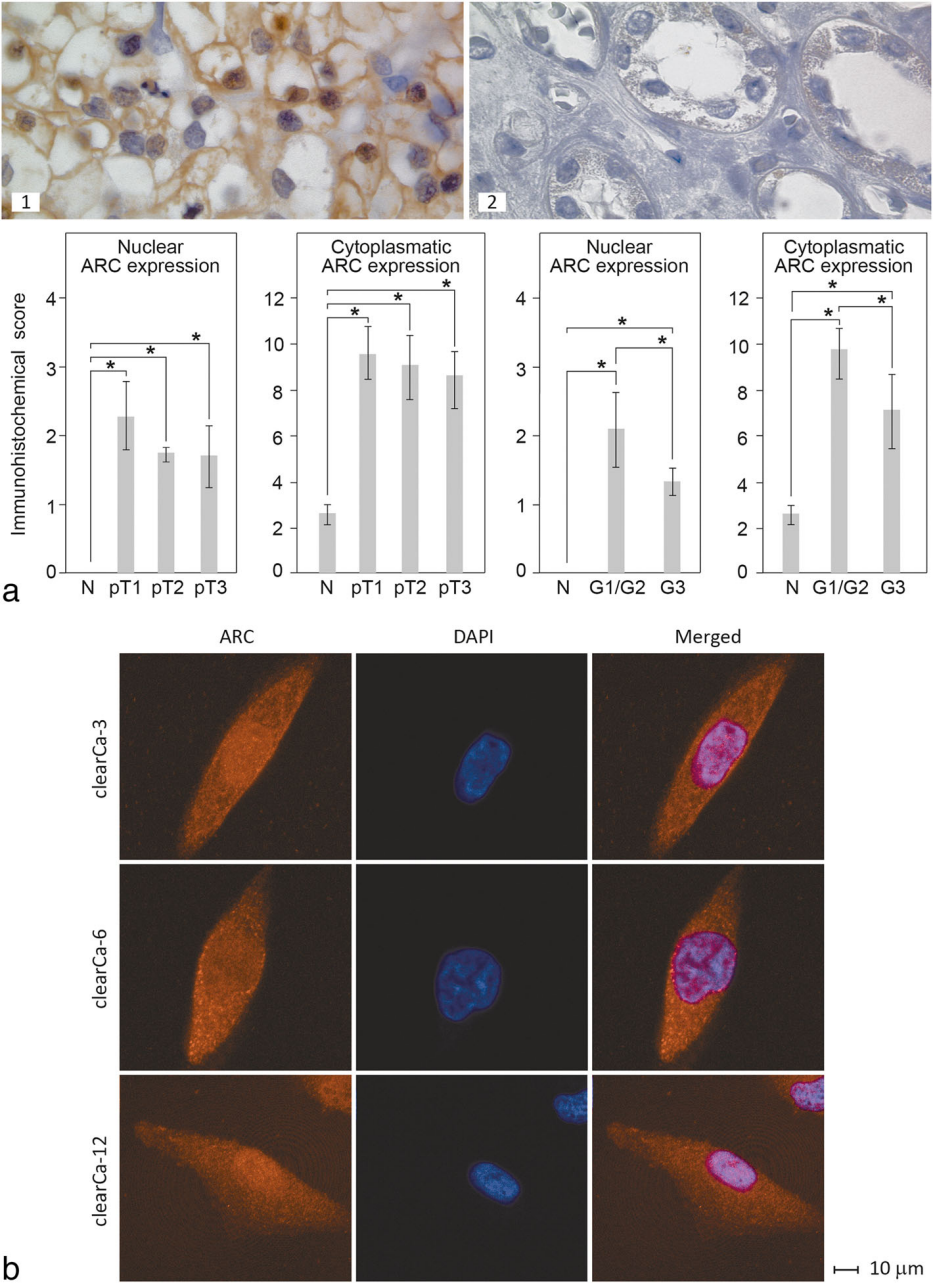
Expression of ARC in clear cell RCCs and RCC cell lines. **a** ARC was strongly overexpressed in the cytoplasm of clear cell RCCs (1) compared to non-neoplastic renal tissue (2), as determined by immunohistochemistry. Furthermore, nuclear ARC expression was only detectable in RCCs and not in non-neoplastic tissue. For semiquantitative analysis of ARC expression cytoplasmatic stainig was scored from 0 to 12 and nuclear expression was scored from 0 to 4. All values are expressed as the mean ± s.d. *p < 0.05. **b** ARC was also strongly expressed in the cytoplasm and nucleus of the RCC cell lines clearCa-6, −3 and −12. Nuclear and cytoplasmic distribution of ARC differed only slightly between the three RCC cell lines, as determined by fluorescent immunohistochemistry

Our statistical analysis of ARC expression in 41 clear cell RCC samples of different tumour stages and grades (Table 2) and 23 corresponding samples of non-neoplastic renal tissue using Student’s *t*-test revealed a significant increase in cytoplasmic ARC expression in RCCs of all pathological stages and grades compared to non-neoplastic renal tissue. However, no significant differences were observed between different tumour stages. In addition, nuclear ARC expression was detectable in all RCCs, whereas none of the non-neoplastic samples demonstrated nuclear ARC expression (Fig. 1a).

**Table 2:** Clear Cell RCC samples

	M	F	G1	G2	G3
N	14	9	1	31	9
pT1	9	6	1	12	2
pT2	6	6	0	9	3
pT3	11	3	0	10	4

Forty one clear cell RCC samples of different tumour stages (pT) and grades (G) and 23 corresponding samples of non-neoplastic renal tissue (N) were used to determine the intensity and subcellular localisation of ARC protein expression by immunohistochemistry. *M* = male, *F* = female

With regard to tumour grades, a slight but significant decrease in nuclear as well as cytoplasmic ARC expression was observed when comparing G1 and G2 with G3 RCCs (Fig. 1a). Based on this result, it is reasonable to assume that ARC expression plays a more important role in apoptosis repression in well and moderately differentiated RCCs compared to poorly differentiated RCCs, which likely possess additional mechanisms of apoptosis resistance.

Our analysis of the subcellular distribution of ARC protein in three RCC cell lines using fluorescent immunohistochemistry revealed that ARC was strongly expressed in the cytoplasm and nuclei, with only minor differences in the distribution pattern between the cell lines (Fig. 1b).

### ARC knockdown neither enhances the induction of p53 inducible genes by topotecan nor induces p53 translocation to the nucleus in RCCs

ARC is strongly expressed in the nuclei of breast cancer cells that express wild-type (wt) p53, and ARC is known to inhibit p53-induced apoptosis via a direct interaction with p53 and its subsequent translocation to the cytoplasm [32]. In RCCs, p53 activation is strongly impaired, although p53 is not mutated in the majority of tumours [33]. Therefore, we tested whether the nuclear expression of ARC in RCCs participates in this functional inhibition of p53.

**Fig. 2:**
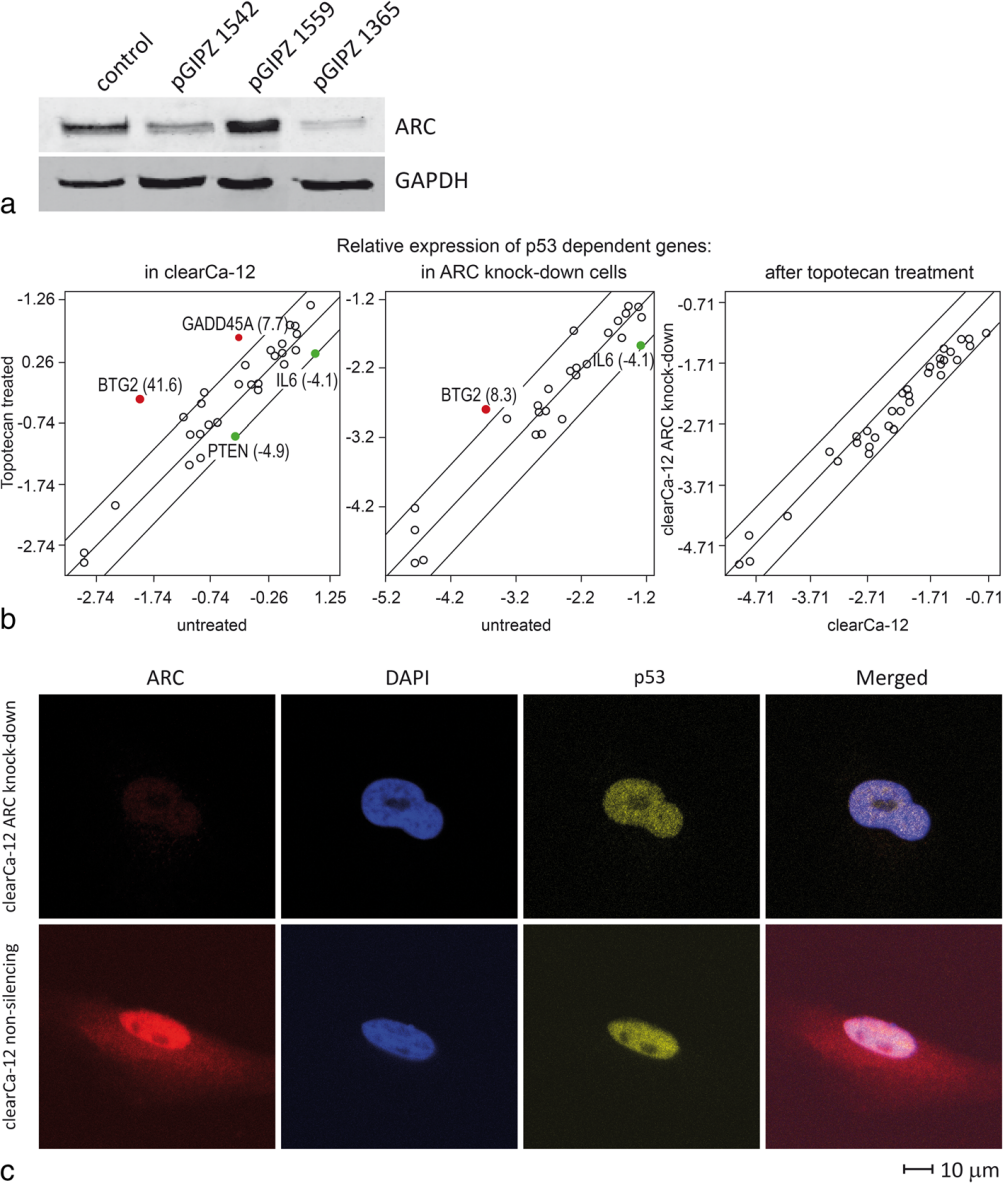
ARC and p53 in RCC cell lines. **a** For ARC knockdown by shRNA in clearCa-12 cells, three lentiviral shRNA vectors were tested, and the shRNA construct pGIPZ 1365 demonstrated the strongest ARC knockdown (approximately 90%) as determined by western blot. **b** Treatment of clearCa-12 cells with 10 μg/ml topotecan modulated gene expression of 4 out of 26 p53-regulated genes in control cells and 2 out of 26 p53-regulated genes in ARC-knockdown cells, as determined using the p53 Signaling Pathway RT^2^ Profiler PCR Array (Qiagen, Hilden, Germany) after 12 h of topotecan treatment. Direct comparison of topotecan treated ARC knockdown and control clearCa-12 cells showed no significant differences with regard to the modulation of p53-regulated genes. Genes with a fold-increase in gene-expression greater than 4 are depicted as red points, and those with a fold-decrease greater than 4 are depicted as green points. **c** ARC knockdown did not change the subcellular localisation of p53 in RCCs. Fluorescent immunohistochemistry for p53 in clearCa-12 cells revealed that ARC knockdown did not change the amount of nuclear p53 in ARC knockdown cells compared to control cells transduced with non-silencing shRNA

As shown in Fig. 2a we performed ARC knockdown in clearCa-12 cells using lentiviral transduction of pGIPZ shRNAmir constructs. As a control, we used the pGIPZ non-silencing shRNA plasmid carrying a scrambled shRNA. Different pGIPZ shRNAmir constructs were tested to reach the strongest knockdown of ARC. For further studies we used cells transduced with the pGIPZ1365 shRNA. ARC knockdown was confirmed by western blotting (Fig. 2a).

We first treated clearCa-12 cells (expressing wt p53) with 10 μg/ml topotecan, a classic DNA-damaging drug, and determined the regulation of p53 target gene expression in comparison to an untreated control using a real time PCR array. This analysis revealed that a change in expression was only observed in 4 of 26 p53 target genes and therefore confirmed that the activation of p53 was impaired (Fig. 2b). Next, we treated ARC-knockdown cells with topotecan (10 μg/ml) and compared the regulation of p53 target genes between these 2 groups. Using this approach, no significant differences in the induction of p53-target genes between ARC-knockdown and control cells were detected (Fig. 2b). These results suggest that nuclear ARC expression in RCCs does not participate in the regulation of p53-dependent gene expression.

Furthermore, comparison of the subcellular localisation of p53 in the ARC-knockdown and control clearCa-12 cells by fluorescent immunohistochemistry showed no differences (Fig. 2c). Thus, ARC knockdown did not result in translocation of p53.

### In addition to ARC, RCCs express anti- and pro-apoptotic Bcl-2 family members of all functional groups

ARC is known to inhibit the mitochondrial pathway of apoptosis by interacting with Bad, Bax and Puma, which are pro-apoptotic members of the Bcl-2 family [18, 19]. Thus, it is reasonable to assume that ARC cooperates with the anti-apoptotic members of the Bcl-2 family in protecting the mitochondria by lowering the availability of these pro-apoptotic Bcl-2 family members. Therefore, we investigated the expression of ARC as well as multiple pro- and anti-apoptotic Bcl-2 family members in 7 RCC cell lines.

**Fig. 3:**
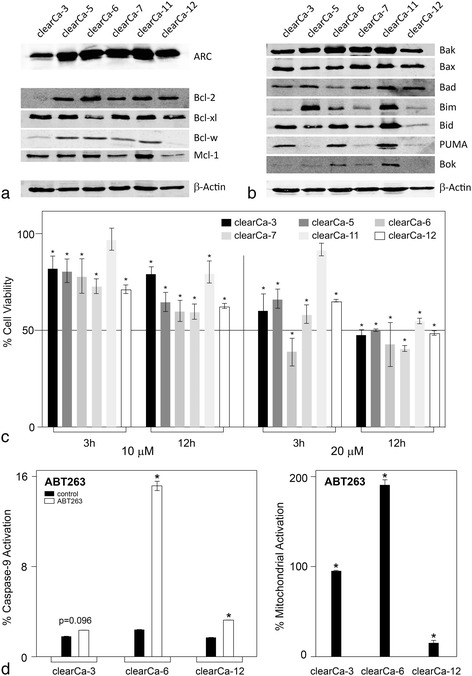
Expression of ARC and Bcl-2 family members and ABT263-induced apoptosis in RCC cell lines. **a** ARC as well as anti-apoptotic Bcl-2 family members were expressed in RCC cell lines, but expression intensity differed between these cell lines. Overall, Bcl-2, Bcl-xl and Mcl-1 exhibited the strongest expression, whereas Bcl-w showed weaker expression. Bcl-2 was not expressed in clearCa-3 cells and Bcl-w was not expressed in clearCa-12 cells. BCL-A1 could not be detected (not shown). The p53 mutational status of the cell lines were published by our group elsewhere [39, 40]. **b** RCC cell lines expressed pro-apoptotic Bcl-2 family members of all functional groups. Sensitisers (Bid, Bim), activators (Puma, Bad, Bok) and effectors (Bax, Bak) were detectable in all RCC cell lines, also with differences in the intensity of expression, with clear expression of PUMA only in clearCa-3, clearCa-6 and clearCa-11 and Bok only in clearCa-6, claerCa-7 and clearCa-11. **c** All RCC cell lines revealed sensitivity towards ABT263-induced cell death (10 and 20 μM ABT263), as determined by cell count. All values are expressed as the mean ± s.d. *p < 0.05. **d** In 3 arbitrarily selected cell lines (clearCa-3, −6 and −12) ABT263 (10 μM) induced mitochondrial apoptosis as determined by caspase-9 and mitochondrial activation (JC-1 stain). All values are expressed as the mean ± s.d. *p < 0.05

The results demonstrate that ARC is stongly expressed in all RCC cell lines, whereas expression of the antiapoptotic Bcl-2 famlily members (Bcl-2, Bcl-xl, Bcl-w and Mcl-1) differs between the cell lines, with a strong expression of Bcl-xl in clearCa-3, −5, −7, and −11 as well as Mcl-1 in clearCa-11 as compared to the other RCC cell lines and a weak expression of Bcl-w in all tested cell lines as well as Bcl-2 and Mcl-1 in clearCa-3 and clearCa-12, respectively (Fig. 3a). On the other hand, multiple pro-apoptotic Bcl-2 family members of all functional groups were expressed [34]: effectors (Bax, Bak), sensitisers (Bid, Bim) and activators (Puma, Bad, Bok). However, especially the expression of the activators differed between the cell lines with a clearly detectable expression of PUMA only in clearCa-3, clearCa-6, and clearCa-11 and Bok only in clearCa-6, clearCa-7, and clearCa-11. Nevertheless, at least one member of each functional group could be detected in each cell line (Fig. 3b).

### RCCs are sensitive towards ABT263-induced apoptosis

The results outlined above propose, that the RCC cell lines could be “primed for death” and we therefore treated them with 10 and 20 μM ABT263, an orally bioavailable Bcl-2 antagonist. In accordance with the results of Zall and co-workers [28], who treated RCCs with ABT737, a closely related compound to ABT263, all RCCs were sensitive to ABT263-induced cell death as determined by cell count (Fig. 3c). The observed reduction in cell number after 3 and 12 h was due to the induction of mitochondrial apoptosis in three arbitrarily selected RCC cell lines, as determined by caspase-9 activation as well as mitochondrial depolarisation (Fig. 3d).

### ARC knockdown sensitises RCC cell lines towards extrinsic (TRAIL-induced) apoptosis

**Fig. 4:**
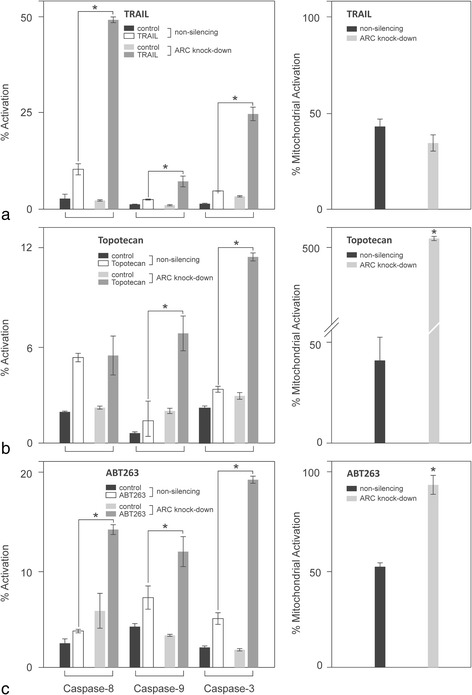
Knockdown of ARC sensitises clearCa-12 towards TRAIL-, topotecan- and ABT263-induced apoptosis. ARC knockdown clearCa-12 or control clearCa-12 cells (non-silencing) were treated with (**a**) TRAIL (100 ng/ml), (**b**) topotecan (10 μg/ml), or (**c**) ABT263 (10 μM). ARC knockdown sensitised clearCa-12 cells against the extrinsic apoptotic pathway induced by TRAIL as determined by caspase-8 and caspase-3 activation. Topotecan and ABT263 induced mitochondrial apoptosis as determined by caspase-9 and caspase-3 activation respectively mitochondrial activation. Furthermore, ARC knockdown enhanced caspase-9 activation induced by TRAIL. All values are expressed as the mean ± s.d. *p < 0.05

To examine the role of ARC in the inhibition of the extrinsic apoptotic pathway in RCCs, we compared the induction of apoptosis following treatment with 100 ng/ml TRAIL in clearCa-12 ARC knockdown cells and control cells expressing a scrambled shRNA. Here, ARC knockdown strongly enhanced TRAIL-induced caspase-8 (and −3) activation, which indicated the importance of ARC for the inhibition of TRAIL-induced extrinsic apoptosis in RCCs. Furthermore, a slight increase in mitochondrial apoptosis, as determined by caspase-9 activation, was also observed upon TRAIL administration (Fig. 4a).

### ARC knockdown sensitises RCC cell lines towards intrinsic (ABT263- and topotecan-induced) apoptosis

To test the hypothesis that ARC cooperates with anti-apoptotic Bcl-2 family members in inhibiting mitochondrial apoptosis, ARC knockdown clearCa-12 cells were treated with topotecan as well as ABT263. Interestingly, ARC knockdown sensitised the RCC cells towards topotecan- (Fig. 4b) and ABT263-induced (Fig. 4c) apoptosis by enhancing the activation of the mitochondrial pathway, as determined by caspase-9 and caspase-3 activation as well as mitochondrial depolarisation. These results confirmed that the strong expression of ARC in RCCs plays an important role in inhibiting intrinsic/mitochondrial apoptosis.

### Topotecan sensitizes RCC cell lines to ABT-263 induced apoptosis

**Fig. 5:**
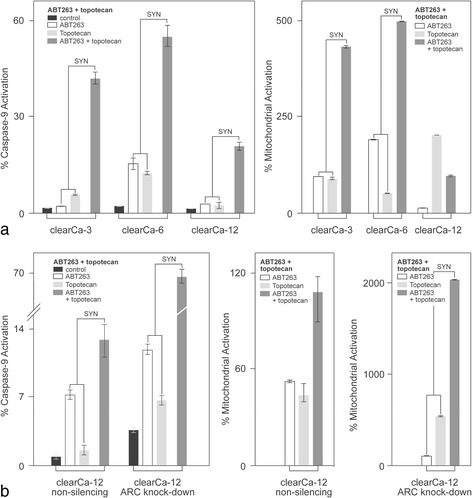
ARC knockdown further enhances topotecan-induced sensitisation towards ABT263-induced apoptosis. **a** ClearCa-3, −6 and −12 cells were treated with 0.1 μg/ml or 10 μg/ml topotecan for 24 h. Co-treatment with 10 μM ABT263 for the last 3 h of the incubation synergistically enhanced caspase-9 activation as well as mitochondrial depolarisation (JC-1) measured by flow cytometry, compared to single treatment with topotecan or ABT263. **b** ClearCa-12 cells transduced with non-silencing shRNA or ARC knockdown shRNA were treated with 10 μg/ml topotecan for 24 h, and 10 μM ABT263 was added for the final 3 h of incubation. ARC knockdown further enhanced caspase-9 activation as well as mitochondrial depolarisation following co-treatment compared to cells transduced with non-silencing shRNA. A synergistic effect (SYN) was determined as i_1,2_ ≥ (i_1_ + i_2_) +20%, \where i_1,2_ = effect of co-treatment, i_1_ = effect of topotecan and i_2_ = effect of ABT263

Next, we treated clearCa-3, −6, and −12 cells with topotecan (0.1 μg/ml or 10 μg/ml) for 24 h and added 10 μM ABT263 for the last 3 h of incubation. In concordance with the observation made by other groups on Etoposide [28], this co-treatment resulted in a synergistic effect and strongly enhanced mitochondrial apoptosis, as determined by mitochondrial depolarisation as well as caspase-9 activation in clearCa-6 cells. A smaller but nevertheless synergistic effect on mitochondrial apoptosis by topotecan pre-treatment could also be observed in the other two cell lines, clearCa-12 and clearCa-3 (Fig. 5a).

### ARC knockdown in combination with topotecan treatment synergistically enhances ABT263-induced apoptosis

Based on the results described above, we hypothesised that a combination of ARC knockdown, which increases the availability of pro-apoptotic Bcl-2 family members, and topotecan treatment, would further enhance ABT263-induced apoptosis. We, therefore, co-treated ARC-knockdown clearCa-12 cells with topotecan and ABT263 as previously described, and this treatment synergistically enhanced ABT263-induced apoptosis in comparison to control clearCa-12 cells (Fig. 5b).

### ARC expression is not regulated by topotecan or UO126

Next, we sought to modify ARC expression using chemical compounds to evaluate possible therapeutic approaches for downregulating ARC.

**Fig. 6:**
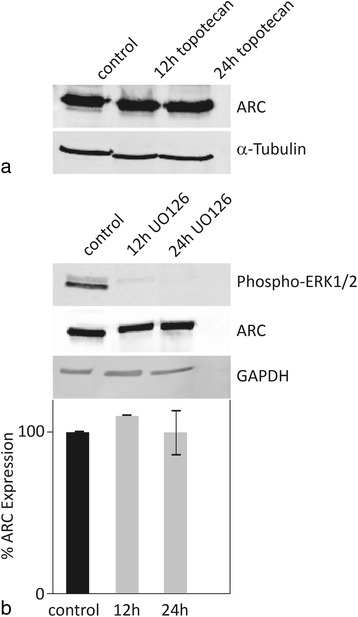
ARC is not regulated by topotecan or UO126. **a** ClearCa-12 cells were treated with topotecan (10 μg/ml) for 12 h and 24 h. At the protein level (as determined by western blot), no change in ARC expression was observed. **b** ClearCa-12 cells were treated with the ERK1/2 inhibitor UO126 (50 μM) for 12 and 24 h. This treatment resulted in nearly the complete loss of ERK1/2 phosphorylation. However, no change in ARC expression could be demonstrated on protein level by western blot analysis

It was previously shown in muscle cells that activation of p53 results in the downregulation of ARC [19]. In clearCa-12 cells, however, treatment with 10 μg/ml topotecan was not able to regulate ARC expression at the protein level (Fig. 6a).

In a previous study using colon cancer cell lines, the activation of ERK by RAS oncogenes was proposed as a mechanism responsible for the strong expression of ARC [35]. Therefore, we treated clearCa-12 cells with the ERK inhibitor UO126 (50 μM) for 12 and 24 h. Although this treatment nearly completely abolished ERK phosphorylation, ARC protein expression was not altered (Fig. 6b).

## Discussion

The resistance of the tumor cells toward apoptosis is a hallmark in many tumors. Combination therapy targeting apoptotic pathways (i.e., Bcl-2 inhibition) is a promising strategy and is currently evaluated in many clinical trials [1]. Despite marked efforts in therapeutic strategies the resistance remains a major problem in cancer therapy. There is a growing interest to find molecular targets by which apoptosis can be selectively induced in tumor cells. One of the promising targets is Bcl-2, against which some inhibitiors have already been approved (i.e., ABT-263, ABT-199) [23]. Bcl-2 inhibitors showed enhanced efficiacy in combination with conventional chemotherapeutic drugs (i.e., paclitaxel, 5-FU, topotecan) but with these combinations still not all of the tumor cells can be reached [23, 24]. For this reason, we focused on ARC protein, which was expressed in all RCC cell lines and tumor samples, we have investigated so far.

This resistance towards mitochondrial apoptosis has mainly been attributed to the anti-apoptotic members of the Bcl-2 family [27, 28, 36, 37]. Cancer cells can evade apoptosis by the up-regulation of the pro-survival Bcl-2 family proteins such as Bcl-2, Bcl-xl, and Mcl-1 [26]. Controversely, Mcl-1 can be up-regulated by ABT-263 which contributes to ABT-263 resistance in cancer cells. ABT-263 increases Mcl-1 stability, but the inhibition of ERK, JNK or Akt activity can sensitise the cancer cells to ABT-263 [38].

The current study was the first to show that ARC, which is overexpressed especially in the cytoplasm of RCCs, strongly participates in this mitochondrial resistance. Therefore, ARC is a functionally relevant anti-apoptotic factor in RCCs acting upstream of the Bcl-2 family members and supporting anti-apoptotic Bcl-2 family members in preventing apoptosis. ARC overexpression could be detected in a couple of cancer types and cancer cell lines and in colorectal cancer cells its expression level is correlated inversely to apoptosis in response to chemotherapy [3–7, 9].

However, our previous study of ARC expression in RCCs did not systematically analyse the subcellular localisation of ARC in RCCs. Here, we refined our analysis of ARC expression in RCCs in vivo with regard to its cellular distribution and found that RCCs express ARC mainly in the cytoplasm, whereas nuclear expression was observed in a much smaller proportion of tumour cells in vivo. ARC is also strongly expressed in the cytoplasm and nucleus of RCC cell lines, with nuclear and cytoplasmic distribution of ARC differing only slightly between three tested cell lines. These results are consistent with the findings of other groups demonstrating strong ARC expression not only in the cytoplasm but also in the nuclei of multiple cancer cell lines [5].

ARC inhibits apoptosis on multiple levels and thereby acts as an upstream apoptosis inhibitor regulating the extrinsic and intrinsic apoptotic pathways in different solid tumours. Regarding the intrinsic pathway of apoptosis, ARC has been reported to prevent p53 tetramerisation [32], inhibit caspase-2 activation [3], and bind pro-apoptotic Bcl-2 family members [18, 19]. With regard to the extrinsic pathway, ARC interacts with caspase-8 [3]. However, the exact role of ARC in the inhibition of apoptosis in RCCs has not been evaluated. Here, we demonstrated that nuclear ARC expression was of only minor importance for the regulation of p53-induced apoptosis in RCCs, as RT-PCR array analysis revealed that knockdown of ARC did not influence the regulation of p53 target genes. Furthermore, the overall regulation of p53 target genes following treatment with a high concentration of topotecan was weak, with only 4 of 26 p53-target genes regulated. These results show that p53 activity is strongly impaired in RCCs [33] and this impaired p53 activation was not due to ARC expression. In contrast to observations made in breast cancer cell lines, ARC knockdown in RCC cells did not result in p53 translocation to the nucleus [14, 32].

In regard to the extrinsic apoptotic pathway, our results demonstrate that ARC plays an important role in the inhibition of TRAIL-induced apoptosis in RCCs, in concordance with other solid tumours. Consequently, TRAIL-mediated caspase-8 and −3 activation were significantly enhanced by ARC-knockdown. Furthermore, ARC-knockdown slightly enhanced mitochondrial apoptosis, providing an initial clue that ARC may also participate in protecting the mitochondria of RCCs against apoptotic stimuli. We conclude that, ARC prevents activation of the apoptotic initiator caspase-8 as well as activation of the mitochondrial amplification loop.

Our results also indicated that ARC plays an important role in the impairment of intrinsic apoptosis: mitochondrial activation was suppressed and controlled by ARC. In contrast, ARC-knockdown sensitised RCC cell lines to mitochondrial apoptosis induced by topotecan and/or Bcl-2 antagonist ABT-263. In case of ARC knockdown, assembly of death-inducing signaling complex (DISC) will be facilitated and spontaneous Bax activation will be triggered resulting in apoptosis [8, 16]. In conclusion, our results suggest that ARC expression in RCCs plays a major role in therapy resistance, even if a targeted drug (i.e., Bcl-2 inhibitor) is given.

The increase in mitochondrial apoptosis upon ARC knockdown was an important finding, as we and others have previously demonstrated that altered mitochondrial activation is crucial for the therapy resistance observed in RCCs [27, 28, 36, 37, 39].

Membrane binded Bcl-2 and Bcl-xl inhibit the release of many apoptotic proteins from mitochondria (i.e., cytochrome c, pro-caspase 3, and apoptosis inducing factor). Bcl-2, which is overexpressed in most RCCs, contributes to tumor development and progression. In addition, Bcl-2 overexpression is correlated with a low apoptosis rate of tumor cells [1]. ARC functions as an anti-apoptotic regulator upstream of Bcl-2 family members by interacting with and thereby reducing the availability of pro-apoptotic binding partners of the Bcl-2 family, including Puma, Bax and Bad. Thus, ARC plays a pivotal role in fine-tuning the apoptotic machinery.

In addition to Puma, Bax, and Bad, all RCC cell lines expressed pro-apoptotic Bcl-2 family members, including Bid, Bim, Bok, and Bak. Although expression levels of these proteins differed between the tested cell lines, members of all functional groups (sensitisers, activators, and effectors) were detectable in each cell line, and therefore, we termed RCC cell lines “primed for death” according to the model of Deng et al. [34]. In summary, these findings provided rationale for the sensitivity of RCCs towards Bcl-2 inhibitors such as ABT263. In accordance with previous observations on ABT-737 and RCCs [28], these “primed for death” cells were all sensitive to ABT-263-induced apoptosis, albeit only to a certain degree. This limited sensitivity of our RCC cell lines towards ABT-263 showed some correlation to the expression profile of anti-apoptotic Bcl-2 family members. In all cell lines evaluated, Mcl-1, which is not inhibited by ABT263 [26], was detected at the protein level. The ABT-263 binding partners Bcl-2, Bcl-w and Bcl-xl were also expressed in these cell lines, with the exception of Bcl-2 in clearCa-3 and Bcl-w in clearCa-12.

Taken together, our findings regarding ARC and Bcl-2 family member expression suggest that the limited sensitivity of RCCs to Bcl-2 inhibitors, which is commonly explained by the presence or absence of pro- and anti-apoptotic Bcl-2 family members themselves [27, 28, 36, 37], also depends on other factors such as ARC interacting with these Bcl-2 family members. These additional factors can modulate the sensitivity of cells towards Bcl-2 antagonists, thereby supporting the role of anti-apoptotic Bcl-2 inhibitors in protecting the mitochondria from apoptotic signals. Moreover, the downregulation of ARC expression likely increased the availability of pro-apoptotic binding partners, including Bax, Bad and Puma, at the mitochondria and thereby enhanced mitochondrial apoptosis.

Due to its important role in inhibiting extrinsic and intrinsic apoptosis, we sought to downregulate the expression of ARC in our RCC cell lines using chemical compounds to evaluate new approaches for targeting ARC overexpression therapeutically. However, our attempts to modulate ARC expression by mechanisms previously described in other cell lines were not successful in RCC cells; neither topotecan, as a classic chemotherapeutic compound [19], nor the ERK-inhibitor UO126 [35] were able to downregulate ARC expression, although both mechanisms were previously described to inhibit ARC gene expression in myocardial and colon cancer cell lines, respectively. Thus, the precise cellular mechanism responsible for the strong ARC expression in RCCs needs to be determined in further experiments.

Next, we tried to further enhance ABT-263-induced apoptosis in RCCs by a pre-treatment with topotecan. Although the synergistic enhancement of ABT263-induced apoptosis by topotecan was strongest in clearCa-6, a synergistic enhancement of mitochondrial apoptosis could also be observed in clearCa-3 and clearCa-12.

As a result, both strategies – indirectly increasing the availability of pro-apoptotic Bcl-2 family members by ARC knockdown as well as topotecan pre-treatment – led to an increased sensitivity of the RCC cell lines towards ABT263-induced apoptosis.

Therefore, it was reasonable to attempt a combination of these strategies to further enhance anti-Bcl-2 treatment sensitivity. In fact, this strategy synergistically enhanced ABT263-induced apoptosis in all cell lines, which suggests that ARC supports the function of anti-apoptotic Bcl-2 family members in preventing mitochondrial apoptosis in RCC cell lines.

## Conclusion

In conclusion, our study demonstrates that the constitutive overexpression of ARC in RCCs may can explain their well-known resistance to multiple therapeutic strategies that are directed against the extrinsic and intrinsic pathways of apoptosis. Thus, targeting the ARC protein may be a promising new therapeutic approach for RCCs. However, future experiments should address how this overexpression could be overcome to reduce the resistance of RCCs.

## Abbreviations

ARC: Apoptosis repressor with CARD
Bad: Bcl-2-associated death promoter
Bak: Bcl-2 homologous antagonist/killer
Bax: Bcl-2-associated X protein
Bcl-2: B-cell lymphoma 2
Bcl-w: Bcl-2-like protein 2
Bcl-xl: B-cell lymphoma-extra large
Bid: BH3-interacting domain death agonist
Bim: Bcl-2-like protein 11
Bok: Bcl-2-related ovarian killer
CARD: Caspase recruitment domain
DMEM: Dulbecco’s modified Eagle’s medium
DMSO: Dimethyl sulfoxide
EDTA: Ethylenediaminetetraacetic acid
FCS: Foetal calf serum
HEK: Human embryonic kidney
JNK: c-Jun N-terminal kinase
Mcl-1: Induced myeloid leukemia cell differentiation protein
PBS: Phosphate buffered saline
PBST: Phosphate buffered saline + Tween
PUMA: p53 up-regulated modulator of apoptosis
RCC: Renal cell carcinoma/cancer
TRAIL: TNF-related apoptosis-inducing ligand
